# A Genome-Wide Pseudogene Map Reveals the Asymmetric Evolution of the A, B, and D Subgenomes in Common Wheat

**DOI:** 10.3390/plants15050818

**Published:** 2026-03-07

**Authors:** Haifeng Zhu, Hao Tang, Yang Li, Ning Yang, Qin Wang, Fan Yang, Hongshen Wan, Wuyun Yang, Jun Li, Zehou Liu

**Affiliations:** 1 Crop Research Institute, Sichuan Academy of Agricultural Sciences, Chengdu 610066, China; 2 Crop Germplasm Innovation and Genetic Improvement Key Laboratory of Sichuan Province, Chengdu 610066, China; 3 Key Laboratory of Wheat Biology and Genetic Improvement on Southwestern China, Ministry of Agriculture and Rural Affairs, Chengdu 610066, China; 4 Biotechnology and Nuclear Technology Research Institute, Sichuan Academy of Agricultural Sciences, Chengdu 610066, China

**Keywords:** *Triticum aestivum*, pseudogenization, parent gene, functional divergence, evolutionary dynamics

## Abstract

Formerly considered nonfunctional “junk DNA,” pseudogenes have been re-evaluated in light of technological advances in bioinformatics and high-throughput sequencing. The limited research to date on pseudogenes in hexaploid common wheat (*Triticum aestivum* L.) is largely confined to individual gene families, thus hindering our understanding of the long-term evolutionary dynamics among the A, B, and D subgenomes. Using the IWGSC RefSeq v2.1 genome assembly, we performed genome-wide identification, classification, and analysis of pseudogenes in wheat, including their distribution, evolutionary history, and parental gene functions. Marked asymmetries in pseudogene abundance, distribution, evolutionary dynamics, and constraints were detected among the subgenomes. The B subgenome harbored significantly more pseudogenes and showed distinct evolutionary patterns compared with the A and D subgenomes. Pseudogenes were strongly associated with transposable elements and peaks in their formation were incongruent with ancient genomic upheavals of wheat ancestral lineages. The parent genes were predominantly enriched in non-core functions and showed tissue-specific expression. The findings provide direct evidence for long-term asymmetric, post-polyploidization evolution in wheat, enhance our understanding of polyploid genome evolution, and offer a methodological framework for research on other genetically complex polyploid crops.

## 1. Introduction

Common wheat (*Triticum aestivum* L.) is a hexaploid species with a genome composition of AABBDD (2*n* = 6*x* = 42). As a crop of global importance for food security, wheat production occupies more than 220 million ha of farmland, with an annual production of almost 750 million tonnes, supplying approximately 15% of the global daily calorie intake. Following the transition from a hunter–gatherer lifestyle to sedentary agrarian societies, common wheat has been pivotal in the rise of civilizations, and through selection, it has been continuously improved to better meet human needs and for adaptation to diverse environments [1]. Its exceptional ecological adaptability and agricultural productivity are largely attributable to the genetic diversity arising from polyploidization events [2].

The evolutionary history of common wheat involves the early divergence of three diploid ancestral lineages (A, B, and D) and two subsequent allopolyploidization events [3]. One polyploidization event approximately 0.8–1.5 million years ago (MYA) involved hybridization between the A subgenome donor *Triticum urartu* (AA) and a close relative of *Aegilops speltoides* (the B subgenome donor, BB), followed by chromosome doubling, which gave rise to a tetraploid ancestor (AABB). The second polyploidization event occurred approximately 8000–10,000 years ago in the Fertile Crescent, where a cultivated tetraploid wheat hybridized with *Aegilops tauschii* (DD) to form modern hexaploid common wheat [1]. The combination of three differentiated subgenomes within a single nucleus creates a dynamic genomic architecture, making wheat an ideal model system for comparative studies of subgenome evolution following polyploidization [4].

Subgenome differentiation and coevolution are integral to understanding the environmental adaptability of common wheat [5]. Polyploidization is the process whereby an organism undergoes whole-genome duplication (WGD), resulting in an increase in the number of chromosome sets. Polyploidization is a widespread evolutionary mechanism and has been a major driver of diversification in the plant kingdom [6]. Following polyploidization, plant subgenomes often exhibit asymmetric functional and evolutionary differentiation; the synergistic coexistence of the A, B, and D subgenomes in wheat is dynamic and leads to functional partitioning and differential evolutionary rates. Coding gene expression bias [7], differential transposon activity [8], epigenetic differences [9], and structural variation [10] provide evidence for asymmetry among the wheat subgenomes. This asymmetry reflects the functional differentiation of subgenomes following polyploidization and endows wheat with remarkable regulatory plasticity, thereby enabling adaptation to diverse environments and selection developmental stages [8]. Pseudogenes, which are gene copies that have lost their original protein-coding function as a result of inactivating mutations, may provide a unique and stable genetic record for tracing long-term evolutionary dynamics [11].

Pseudogenes arise primarily by three core mechanisms [12]: (1) DNA-mediated duplication, encompassing processes such as unequal crossing over and polyploidization, which generates functionally redundant paralogs that subsequently accumulate degenerative mutations; (2) RNA-mediated retrotransposition, by which reverse-transcribed mRNAs are integrated randomly into the genome, often lacking regulatory elements and hence cannot be expressed, and ultimately result in degeneration; and (3) direct degeneration of functional genes following the relaxation of selective pressure triggered by changes in the genetic background or environmental conditions. The prevalence of pseudogene types generated by these distinct pathways varies among species. For instance, processed pseudogenes—derived from retrotransposition—are abundant in mammalian genomes, but are substantially less prevalent than unprocessed (duplicated) pseudogenes in plants [13,14,15,16]. Owing to the loss of their ancestral protein-coding function, pseudogenes are frequently overlooked in conventional plant genome annotation and subsequent functional studies [17].

The conventional perspective that pseudogenes are nonfunctional has been revised in light of evidence that some pseudogenes remain functional and participate in gene expression regulatory networks [18]. Pseudogenes whose biological functions are poorly characterized may contain information on various aspects of genomic evolution differing from that derived from functional loci [14,19]. This dual characteristic, combining the properties of neutral markers with potential regulatory functions, means that pseudogenes serve as “testing grounds” for genomic innovation: they can accumulate mutations without compromising biological fitness, while their quasi-gene architecture facilitates functional reactivation and the acquisition of novel functions [17,20]. Although the value of pseudogenes in evolutionary research has been directly or indirectly validated in diverse plants, including Arabidopsis and rice [13,16,21,22,23,24], information on pseudogenes at the whole-genome level in common wheat remains limited.

Unlike previous studies of wheat pseudogenes that focused on individual or several specific genes [25,26,27], this study performed a genome-wide identification of pseudogenes using the IWGSC RefSeq v2.1 reference genome. We analyzed the pseudogenes for their distribution, evolutionary dynamics, and divergence from parent genes, and examined the functional differentiation and biases of the parent genes across distinct pseudogene types. This study aimed to elucidate the genome-wide evolutionary dynamics of pseudogenes and the asymmetric evolution of the three subgenomes. Our findings offer a technical framework for further pseudogene research in wheat and serve as a reference for similar genome-wide studies in other plant species.

## 2. Results

### 2.1. Genome-Wide Identification and Classification of Pseudogenes

We identified 79,285 pseudogenes in the wheat genome. The number of different pseudogene types varied substantially. Unitary pseudogenes (Unitary, UNI) and fragmented pseudogenes (FRAG) were the predominant types, comprising 29,237 and 23,394 members, respectively, and accounting for 36.88% and 29.51% of the total pseudogenes, respectively. This result suggests that pseudogene formation in wheat has been primarily driven by gene degeneration and fragmentation. Based on alignment with the parent genes, the number of truncated pseudogenes was significantly lower than that of full-length (FULL) pseudogenes (Supplementary Table S1).

Localized duplication was the primary mechanism of origin detected for duplication-derived pseudogenes (DUP). A total of 7883 DUP pseudogenes (including DUP-full and DUP-truncated) were identified, representing 9.94% of the total number. Chromosomal location association analysis showed that 1598 pseudogenes (2.02%) were located on the same chromosome as their parent genes. A total of 13,985 processed pseudogenes (Retropseudogenes, RET), including RET-full and RET-truncated, were identified, accounting for 17.64% of the total pseudogenes. This result suggests that pseudogene formation in wheat has been primarily driven by gene degeneration and fragmentation, a pattern consistently observed across all three subgenomes.

### 2.2. Genome-Wide Distribution Characteristics of Pseudogenes

The genomic distribution of pseudogenes differed at the subgenome, genomic region, and chromosome levels.

**Figure 1:**
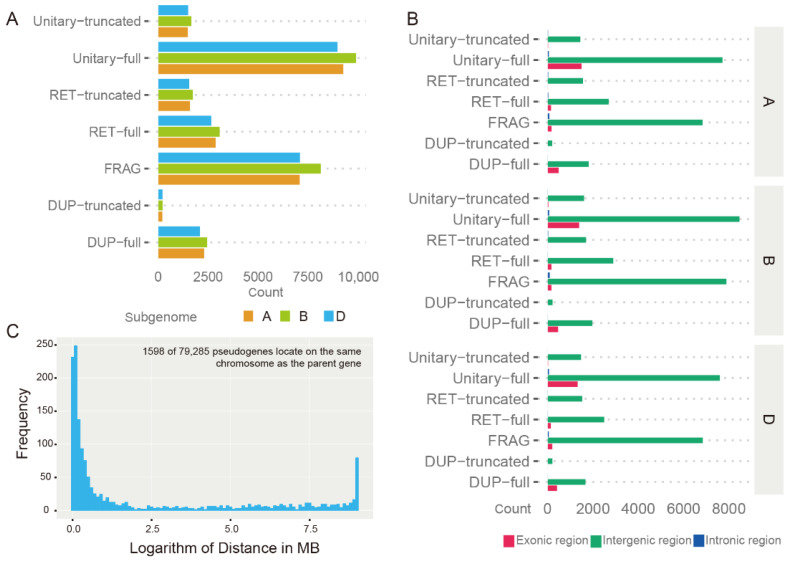
Genome-level Characteristics of Pseudogene Distribution in Common Wheat. (**A**) Total numbers of each pseudogene type in the A, B, and D subgenomes. (**B**) Proportion of pseudogenes located in intergenic regions, introns, and exons. (**C**) Distribution of physical distances between pseudogenes and their parent genes located on the same chromosome. The *x*-axis represents physical distance values transformed by log_2_(Mb + 1); the *y*-axis represents the number of pseudogenes in the corresponding distance intervals.

At the subgenome level, the total number of pseudogenes in the B subgenome (27,187) exceeded that in the A (24,794) and D (24,078) subgenomes. Nevertheless, the proportion of each pseudogene type was relatively consistent across the subgenomes (Figure 1A). Notably, significant differences in physical length were observed among the A, B, and D subgenomes of common wheat. We therefore calculated the subgenome length-corrected pseudogene density, defined as the number of pseudogenes per megabase (Mb) of genomic sequence. The results showed that the pseudogene densities of the A, B, and D subgenomes were 4.98, 5.18, and 6.02 per Mb, respectively.

Regarding genomic region distribution, pseudogenes were strongly concentrated in intergenic regions, accounting for as much as 90.7% of the total, whereas those located in intronic regions and exonic regions each accounted for less than 1% of the total number (Figure 1B). This distribution pattern directly reflected the strength of functional constraints in the different genomic regions. Pseudogene insertion into introns may disrupt splicing signals or the regulation of gene expression, and may lead to their rapid elimination by natural selection; thus, pseudogenes may be highly unstable in intronic regions [28].

**Figure 2:**
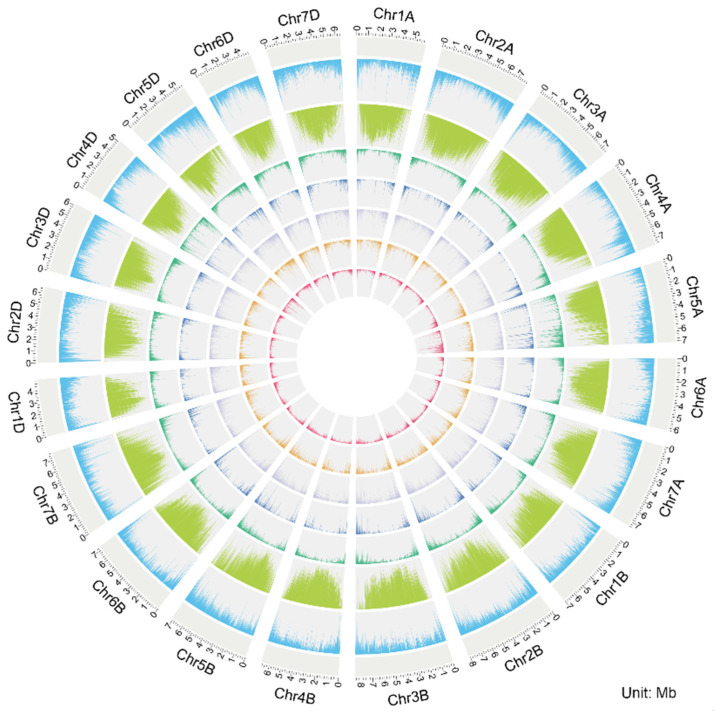
Genome-wide Distribution of Pseudogenes in Common Wheat. Chromosomes are arranged by subgenome (Chr1A to Chr7D). From the outermost to the innermost, the rings represent functional gene density (sky blue), transposable element density (light green), total pseudogene density (green), and the density distribution of four pseudogene types: duplication-derived (DUP, blue), fragmented (FRAG, light purple), processed (RET, orange), and unitary (UNITARY, red). All density values were calculated based on a 1 Mb sliding window and are expressed as the percentage of covered base pairs.

The pseudogene distribution at the chromosomal level showed complementarity to and association with the distribution of functional genes and transposable elements (TEs). The density of functional genes was significantly higher in terminal/subtelomeric regions than in pericentromeric regions. In contrast, TEs were concentrated in pericentromeric regions, showing distinct complementarity with the distribution of functional genes, which is consistent with the general structural characteristics of wheat chromosomes [26]. The majority of pseudogenes (especially the DUP and FRAG types) were distributed consistently with functional genes, i.e., concentrated in the gene-dense terminal regions, reflecting their derivation by duplication or mutation of functional genes. Notably, the distribution of processed pseudogenes (RET) strongly overlapped with TE-rich regions, reflecting the influence of retrotransposition on the formation of this pseudogene type (Figure 2).

### 2.3. Evolutionary Dynamics of Pseudogenes

To investigate subgenomic differentiation in wheat following polyploidization, we performed a one-to-one single-copy syntenic orthologous element analysis. The majority of these orthologous elements (17,720, 61.66%) remained functional in all three subgenomes, suggesting that strong purifying selection is an evolutionary constraint on the wheat genome as a whole. Nevertheless, a substantial number of elements (11,018, 38.34%) showed gene absence (null) or pseudogenization (pseudo) in at least one subgenome. These characteristics exhibited significant evolutionary asymmetry among the three subgenomes. Gene absence or pseudogenization events in the B subgenome totaled 5390, accounting for 36.1% of all variation events (14,945). This proportion was higher than those for the A subgenome (5747, 35.3%) and the D subgenome (4610, 26.6%). The frequency of gene absence and pseudogenization events in the B subgenome was significantly higher than that in the D subgenome (*p* < 0.001), but not significantly different from that in the A subgenome (*p* = 0.36). Collectively, these results indicate that the B subgenome shows relatively high genetic instability, although not statistically significantly higher than that of the A subgenome (Supplementary Table S2).

**Figure 3:**
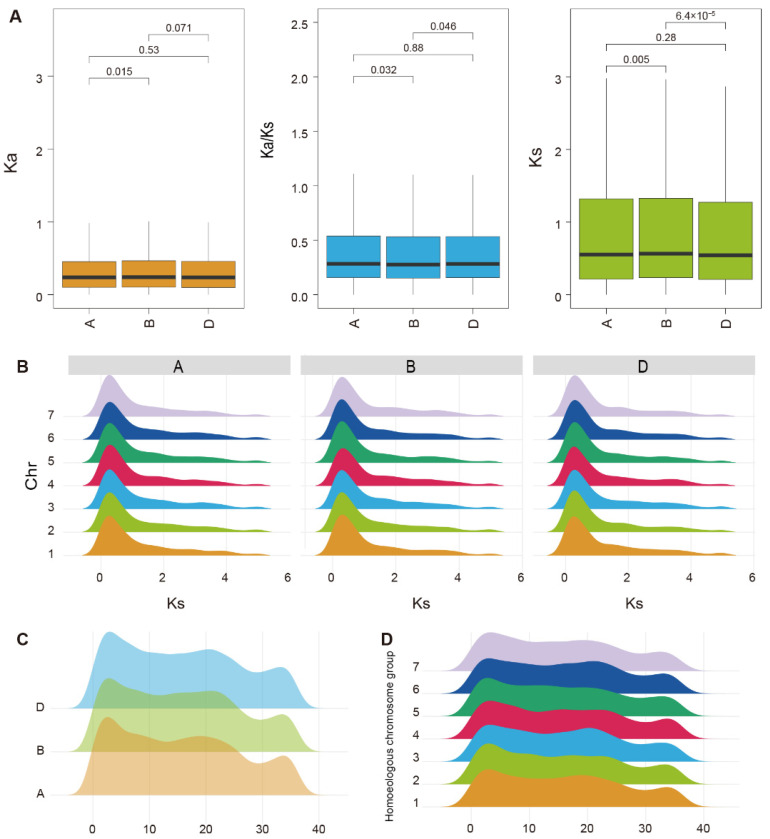
Core Evolutionary Indices Analysis of Wheat Pseudogenes. (**A**) Selection pressure-related indices for pseudogenes across the three subgenomes, comprising the nonsynonymous substitution rate (*K*_a_), synonymous substitution rate (*K*_s_), and their ratio (*K*_a_/*K*_s_). (**B**) *K*_s_ values for pseudogenes for each chromosome across all subgenomes. (**C**) Temporal distribution of pseudogene formation in individual subgenomes (Unit: MYA). (**D**) Temporal distribution of pseudogene formation across individual chromosomes (Unit: MYA).

The *K*_a_ values for the three subgenomes were consistently low with minimal variation, although the values differed significantly between subgenomes A and B (*p* = 0.015), and were marginally significantly different between subgenomes A and D (*p* = 0.071). Similarly, the *K*_s_ values were low with limited variation among the subgenomes, although the differences between subgenomes A and B (*p* = 0.005) and between subgenomes A and D (*p* = 6.4 × 10^5^) were highly significant. The *K*_a_/*K*_s_ ratios for pseudogenes across the three subgenomes were all less than 0.5 (Figure 3A). Comparisons between subgenomes revealed significant differences between subgenomes A and B (*p* = 0.032) and between subgenomes A and D (*p* = 0.046), whereas the difference between subgenomes B and D was non-significant (*p* = 0.88).

The *K*_s_ distribution patterns across chromosomes were highly consistent in subgenomes A, B, and D, suggesting that pseudogenization events occurred synergistically across the three subgenomes. The *K*_s_ values were predominantly in the range of 0–1, with a median of 0.69, which was lower than the mean (2.71), thus representing a right-skewed distribution (Figure 3B).

Temporal distribution curves for pseudogene formation exhibited a distinct three-peak pattern for each subgenome, with strong consistency in the timing of the peaks among the subgenomes (Figure 3C,D). The first peak, which showed the smallest inter-subgenome variation, was concentrated at 3.24–3.68 MYA. The first peak for subgenome A (3.24 MYA) was slightly earlier than those for subgenomes B (3.64 MYA) and D (3.68 MYA). The second peak spanned 18.78–20.18 MYA, which represented the greatest inter-subgenome variation among the three peaks. The third peak displayed strong overlap at 32.31–32.91 MYA. Thus, the timing of pseudogene formation showed considerable evolutionary conservation.

The temporal distribution curves showed a broadly consistent trend at the chromosome level. Pseudogene formation was primarily concentrated in the 0–40 MYA interval. This conserved temporal pattern across chromosomes suggests that pseudogene generation underwent synchronous changes during wheat evolution (Figure 3D).

### 2.4. Comparative Features of Pseudogenes and Their Parent Genes

**Figure 4:**
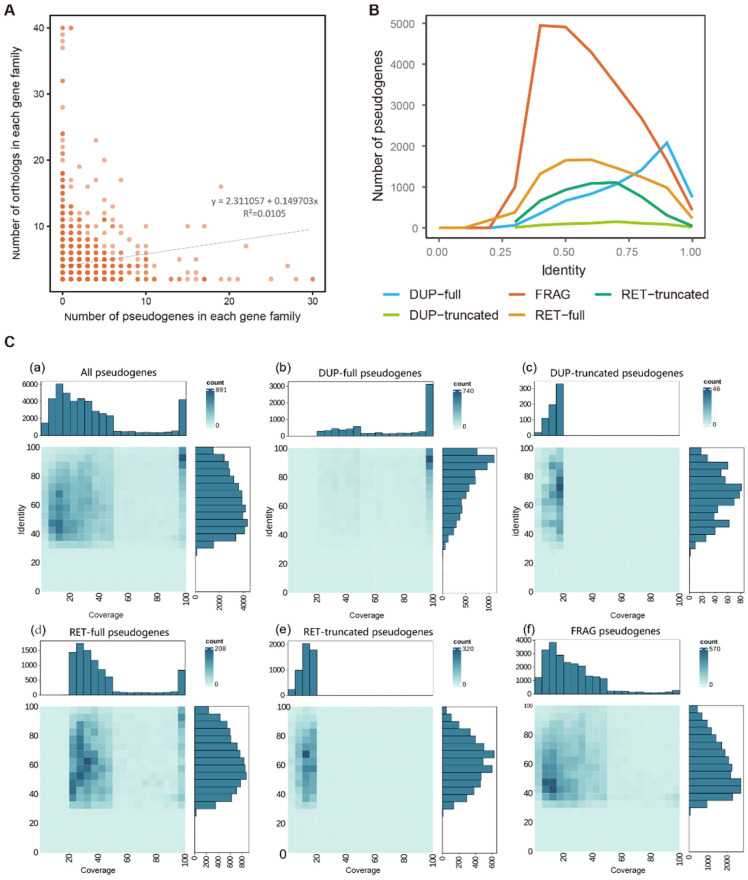
Comparative Analysis of Pseudogenes and Their Parent Genes. (**A**) Distribution of number of pseudogenes versus corresponding number of parent genes in each gene family. (**B**) Distribution of sequence identity for each pseudogene type and their parent genes. (**C**) Comparison of sequence coverage and sequence identity among the pseudogene types and their parent genes: (**a**) all pseudogenes, (**b**) full-length duplicated (DUP-full) pseudogenes, (**c**) truncated duplicated (DUP-truncated) pseudogenes, (**d**) full-length processed (RET-full) pseudogenes, (**e**) truncated processed (RET-truncated) pseudogenes, and (**f**) fragmented (FRAG) pseudogenes.

The number of pseudogenes and the size of the corresponding parent gene families showed a highly significant, weakly positive correlation (Spearman’s rank correlation analysis; *ρ* = 0.1901, *p* < 0.001). This indicates that the larger gene families in wheat tended to accumulate greater numbers of pseudogenes. The weak correlation suggests that specific gene families may have distinct pseudogenization propensities, and that pseudogene formation is also regulated by other biological processes (Figure 4A).

The sequence identity between pseudogenes and their parent genes reflects the degree of evolutionary divergence. Significant differences in sequence identity were detected among different pseudogene types. Note that Unitary pseudogenes were excluded from this analysis because they originate from mutations in single-copy genes with the loss of the parent gene copy, thus precluding sequence alignment. This finding suggests that different pseudogene types have undergone distinct evolutionary trajectories. The majority of pseudogenes exhibited > 0.25 sequence identity with their parent genes, with the peak sequence identity concentrated in the 0.3–0.6 range. Among the pseudogene types, DUP-FULL pseudogenes were over-represented in high-identity intervals, whereas FRAG pseudogenes showed notable enrichment in the 0.7–0.8 sequence identity range (Figure 4B).

Significant differences in sequence coverage were observed among the pseudogene types (Figure 4C). Full-length types (DUP-FULL and RET-FULL) showed the highest coverage, truncated types (DUP-truncated and RET-truncated) had lower coverage, and fragmented pseudogenes (FRAG) showed the lowest coverage. Most pseudogenes showed 0–50% coverage and 40–80% identity, consistent with the pattern exhibited by the FRAG pseudogenes (Figure 4C(f)). The DUP-derived pseudogenes exhibited higher coverage and higher sequence identity than processed pseudogenes (RET; Figure 4C(b,d)). This finding is consistent with the “dead-on-arrival” hypothesis, which proposes that processed pseudogenes rapidly accumulate mutations after their origin, leading to faster divergence from their parent genes compared with DUP pseudogenes [29].

### 2.5. Functional Divergence of Parent Genes Among Pseudogene Types

**Figure 5:**
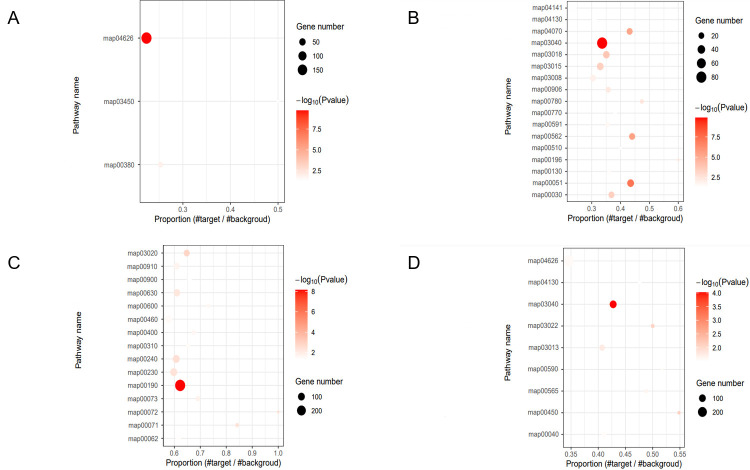
KEGG Pathway Enrichment Analysis of Parent Genes for Each Pseudogene Type in Common Wheat. (**A**) Duplicated pseudogenes, (**B**) processed pseudogenes, (**C**) unitary pseudogenes, and (**D**) fragmented pseudogenes.

Gene ontology (GO) and Kyoto Encyclopedia of Genes and Genomes (KEGG) enrichment analyses revealed the potential biological functions of the parent genes corresponding to each pseudogenes type. For example, the parent genes of DUP pseudogenes were significantly enriched in molecular functions related to basic biological interactions and energy-associated binding in GO enrichment analysis, such as ADP binding (GO:0043531) and protein binding (GO:0005515) (Supplementary Table S3). The corresponding KEGG pathway enrichment analysis showed that these parent genes were significantly enriched in the plant–plant pathogen interaction pathway (map04626) (Figure 5A), indicating that the parent genes are involved in biological stress defense responses.

In contrast, the KEGG pathway enrichment analysis of the parent genes for RET pseudogenes revealed that the spliceosome pathway (map03040), fructose and mannose metabolism (map00051), and inositol phosphate metabolism (map00562) were the most significantly enriched. Notably, the parent genes of FRAG pseudogenes shared similar functional characteristics with those of RET pseudogenes and were significantly enriched in the spliceosome pathway (map03040). This finding indicates that RET and FRAG pseudogenes originated from parent genes involved in post-transcriptional regulatory processes (Figure 5B,D).

The parent genes of Unitary pseudogenes were significantly enriched in the oxidative phosphorylation pathway (map00190) (Figure 5C), which is critical for cellular energy production.

These results demonstrated distinct functional divergence among the parent genes of each pseudogene type, each corresponding to distinct core biological processes.

### 2.6. Characteristics of Conserved Domains in Pseudogenes and the Functional Bias of the Parent Genes

The conserved domains retained by the pseudogenes, which are important molecular imprints of the functions of their parent genes, can further corroborate and improve understanding of the origin and functional preferences of wheat pseudogenes from a sequence structure perspective [30]. Proteins deduced from the pseudogene sequences were annotated based on 1783 Pfam conserved domains (Supplementary Table S4). High-frequency domains (those with occurrence counts ≥500) were mainly associated with three major functional categories. First, a large proportion corresponded to proteins related to mobile genetic elements, such as reverse transcriptases (e.g., PF13456; 3792), integrases/transposases (e.g., PF00665; 1162), and Gag proteins (e.g., PF03732; 649). Domains directly linked to transposition activity accounted for approximately 62% of all annotations. Second, many domains were associated with disease resistance and immune signaling, including NB-ARC (PF00931; 1893), protein kinase (PF00069; 1907), and leucine-rich repeat (PF13855; 1498) domains, which are primarily associated with plant-specific pathogen recognition and defense pathways. Finally, an important category included transcriptional and developmental regulatory proteins, such as those containing FAR1 (PF03101; 1035), PPR (PF01535; 857), and BTB/POZ (PF00651; 716) domains, which mediate the precise regulation of gene expression.

Notably, the intra-species parent genes of these pseudogenes generally exhibited significant tissue-specific expression patterns (Supplementary Figure S1). This expression characteristic reflects the functional attributes of the aforementioned domains, collectively suggesting that the pseudogenization process in common wheat mainly affected “non-core” functional genes that function under specific spatiotemporal conditions.

## 3. Discussion

### 3.1. Dominant Pseudogene Types and Transposition-Driven Mechanism of Origin

This study represents the first genome-wide systematic analysis and classification of pseudogenes in common wheat. Specifically, we identified 24,794, 27,187, and 24,078 pseudogenes in the A, B, and D subgenomes, respectively (Table S1, Figure 1A). Unitary and FRAG pseudogenes are the dominant types in wheat, which is consistent with reports of abundant short gene fragments in closely related species, such as barley [17]. Although Unitary and FRAG pseudogenes predominate in the extant genome, conserved domain analysis revealed that approximately 62% of pseudogene sequences contained transposition-related domains (e.g., reverse transcriptases and integrases) (Supplementary Table S4). However, the mechanisms of pseudogene origins were inferred by retrospective inference based on the sequence characteristics, from which the pseudogenes were classified, rather than classifying the pseudogenes directly based on their mechanism of origin. Transposable element insertions are prevalent in the promoters, UTRs, introns, and exons of wheat genes, with 95.0% of the genes co-localizing with transposons, which are core drivers of pseudogenization [31,32]. Transposon activity provides abundant materials for pseudogene formation by generating incomplete gene copies or disrupting functional genes via insertions [33]. Notably, some gene fragments captured by TEs may be misclassified as FRAG pseudogenes owing to their short length or continuous sequence deletions; in contrast, some genes impacted by TE insertions may be misclassified as Unitary pseudogenes because loss of function occurs through point mutations without large-scale sequence deletions. During the degeneration of pseudogene sequences, non-conserved regions (e.g., functional domains) are prone to mutation or deletion, whereas TE-related domains are more likely to be conserved owing to methylation-mediated protection and sequence conservation [34,35]. This explains why transposition-related domains can still be detected even when the main functional sequences of pseudogenes are completely degenerated.

This transposition-dominated mechanism of origin directly explains the genomic distribution characteristics of pseudogenes. In the present study, *cis*-localized pseudogenes (i.e., those located on the same chromosome as their parental genes) account for only 2.02% of the total number. Pseudogene formation in wheat relies on the reverse transcription function of TEs (e.g., LINE-1), leading to the random insertion of newly generated pseudogenes into non-ancestral loci. In addition, extensive genomic rearrangements that have occurred during wheat polyploidization have further exacerbated the physical separation between pseudogenes and their parental genes [25,33,36].

### 3.2. Evolutionary Asymmetry of Subgenomic Pseudogenes

Compared with its diploid and tetraploid progenitors, common wheat exhibits greater genomic plasticity and broader adaptability, which is partially attributable to the generation of novel genetic diversity following allopolyploidization [37]. The present results showed that the proportions of pseudogene types were relatively consistent among the A, B, and D subgenomes, whereas significant differences were observed in pseudogene abundance and the evolutionary dynamics revealed by synteny analysis. Specifically, the B subgenome harbors the highest number of pseudogenes (27,187), with higher frequencies of gene loss (12.35%) and pseudogenization events (12.53%) compared to the other two subgenomes—significantly so relative to the D subgenome, though with no statistical difference from the A subgenome (Figure 1A, Supplementary Table S2). Notably, the normalized pseudogene density per megabase of genomic sequence was highest in the D subgenome, reflecting a more compact distribution of pseudogenes in this shorter subgenome.

Analysis of evolutionary selective pressure revealed that the *K*_a_/*K*_s_ ratio for the B subgenome was slightly lower than those of the A and D subgenomes (Figure 3A). This unique evolutionary pattern may reflect the earlier divergence of its donor species (≈7 MYA) and potential outcrossing background [38]. These historical factors have promoted the incidence of gene duplication events in the B subgenome, increased its sequence diversity, and a larger number of functionally redundant genes have accumulated. These redundant gene copies provided “raw materials” for pseudogenization, thus explaining why the B subgenome retains the largest number of pseudogenes.

Although pseudogenes are generally considered to evolve neutrally [12], the present findings showed that the *K*_a_/*K*_s_ ratios of pseudogenes across all subgenomes were much lower than 1, consistent with reports for rice and barley [13,17,39]. This phenomenon may be explained by two scenarios: first, except for a few cases of annotation bias (potentially functional genes misannotated as pseudogenes), the integrity of the open reading frame is critical for potential “non-coding functions” of pseudogenes, which are thus subject to moderate purifying selection [30,40]; and second, these pseudogenes may have been subject to strong purifying selection as functional genes for a prolonged evolutionary period before recently undergoing pseudogenization [21,41].

### 3.3. Temporal Dynamics of Pseudogene Formation and Association with Ancient Genomic Events

To clarify the evolutionary origin and temporal dynamics of pseudogenes in wheat, we estimated their formation times, which showed three temporal peaks in pseudogenization (3.24–3.68, 18.78–20.18, and 32.31–32.91 MYA). These peaks are inconsistent with the two known allopolyploidization events in common wheat (0.8–1.5 MYA and 8000–10,000 years ago) (Figure 3C,D). Thus, massive pseudogene accumulation is not a direct consequence of recent polyploidization but is a delayed, selective long-term process; polyploidization primarily releases purifying selection on redundant genes, laying the evolutionary foundation for subsequent pseudogenization [42].

The three ancient peaks may record genomic upheavals experienced by ancestral lineages during geological periods. The earliest peak (≈32–33 MYA) may be associated with the ancient polyploidization event (rho WGD, 98.2 MYA) during the early evolution of the Poaceae lineage and its prolonged diploidization process [43]. the middle peak (≈18–20 MYA) may result from gene duplication cluster events within the Pooideae subfamily (≈33 MYA) [44]; the most recent peak (≈3–4 MYA) may coincide with the key hybridization stage after the divergence of the A and B subgenomes from their common ancestor (6.5 MYA) and before the formation of modern common wheat [45]. These associations suggest that pseudogenes in the current wheat genome are “genomic fossils” of its long evolutionary history, with their formation more closely linked to ancient genome duplication and differentiation events.

Notably, the low median Ks value (0.69) of pseudogene-parent orthologous pairs (Figure 3A,B) seems contradictory to the ancient formation times. Two plausible explanations exist for this. First, the molecular clock evolutionary rate used in the present study may be higher than the actual evolutionary rate for these pseudogenes, leading to systematic overestimation of the absolute formation times. Second, as discussed earlier in this article, pseudogenization is not an absolute functional endpoint—the pseudogene sequences may not evolve entirely neutrally and purifying selection may partially constrain the accumulation of synonymous substitutions (*K*_s_) [30,40]. Thus, calibration of the mutation rate (μ) using species-specific divergence events and other approaches is required in future studies.

### 3.4. Which Genes Are More Susceptible to Pseudogenization?

Functional selectivity is a core characteristic of pseudogenization. The parent genes of pseudogenes are significantly enriched in functional categories related to environmental adaptation, including defense responses, cell wall modification, secondary metabolism, and protein degradation (Supplementary Figure S1; Supplementary Table S1 for GO/KEGG enrichment results). These parent genes are predominantly derived from highly redundant gene families and exhibit distinct tissue-specific expression patterns.

This functional selectivity is consistent with findings reported for Arabidopsis, rice, and other plant species [21], and reflects a conserved rule throughout the plant kingdom: core housekeeping genes (e.g., those involved in DNA replication and energy metabolism) are constrained by strong purifying selection, thus maintaining high sequence conservation and a low likelihood of pseudogenization. In contrast, environment-related genes evolve more rapidly, undergo more frequent variation in copy numbers, and exhibit greater tolerance to functional loss, and thus are primary targets for pseudogenization. This selective pattern alleviates the genomic functional burden and preserves the evolutionary flexibility among functional genes, providing a foundation for genomic plasticity that confers adaptability to complex environmental and artificial selection pressures.

## 4. Materials and Methods

### 4.1. Genomic and Transcriptomic Dataset Collection

All genomic sequences and annotations were downloaded from public databases.

This study utilized common wheat Chinese Spring (CS) as the reference genome, owing to its well-annotated genome and abundant genetic and molecular resources [46]. The core dataset was the IWGSC RefSeq v2.1 reference genome sequence [47] (https://www.wheatgenome.org, accessed on 4 March 2026), supplemented with data from four public databases: (1) SWISS-PROT [48] (https://www.sib.swiss/swiss-prot, accessed on 4 March 2026) for protein sequence data; (2) Repbase, a database of genomic repetitive sequences [49] (https://www.girinst.org/repbase, accessed on 4 March 2026) for retrieving transposable element (TE) sequences; (3) the Pfam database [50] (https://pfam.xfam.org, accessed on 4 March 2026) for annotating conserved protein domains; and (4) TriticeaeExpDB (https://triticeaeexpdb.cn, accessed on 4 March 2026), which provides expression levels of parent genes of pseudogenes across different tissues and developmental stages.

### 4.2. Construction of the Reference Protein Sequence Set

To ensure the accuracy of pseudogene identification, we integrated two protein datasets to construct a reference sequence set in this study: (1) Dataset 1 comprised proteins from the target species and its closely related homologs including *Triticum urartu*, *Triticum turgidum*, *Aegilops tauschii*, *Aegilops speltoides*, *Thinopyrum elongatum*, Secale cereale and Hordeum vulgare, with the following selection criteria: intact start and stop codons, functionally validated via alignment against SWISS-PROT, and exclusion of transposon-associated proteins following alignment with the RepBase database; (2) Dataset 2 was retrieved directly from the SWISS-PROT database, excluding fragmented sequences annotated as “fragment” in RepBase and transposon-related proteins. After merging these two datasets, CD-HIT [51] was employed for sequence deduplication, and non-redundant reference protein sequences were ultimately obtained.

### 4.3. Pseudogene Identification and Classification

Pseudogene identification was performed via a three-step strategy. First, high-quality Coding Sequence (CDS) sequences—extracted from the reference genome after hard-masking repetitive regions—were aligned against the reference protein set using the BLASTX algorithm implemented in DIAMOND v0.9.24.125 [52]. Second, candidate pseudogene loci were extracted: Genomic regions aligned to the same protein were merged, with 2-kbp extensions upstream and downstream to define candidate loci. For overlapping genomic regions aligned to distinct reference proteins, the optimal alignment was retained according to bit scores. Third, Genewise (wise2-4-1) [53] was employed to perform precise alignment between each candidate locus and its corresponding reference protein, enabling the detection of characteristic features of pseudogenes, including frameshift mutations, premature stop codons, abnormal introns, incomplete coding regions, and absence of promoters/regulatory regions. This step ultimately yielded the final set of identified pseudogenes.

Pseudogenes were classified into four categories based on sequence characteristics and alignment results: Duplicate Pseudogenes (DUP), Unitary Pseudogenes (Unitary, UNI), Processed Pseudogenes (retropseudogenes, RET), and Fragmented Pseudogenes (FRAG). First only sequences with an identity greater than 30% can be identified as pseudogenes, as this threshold is the established standard for reliable homology inference, effectively eliminating false-positive random matches while retaining bona fide pseudogene loci of homologous origin [54]. Among these, FRAG pseudogenes were defined as those that align exclusively to a single CDS region of a multi-exon parent gene and do not span exon-intron boundaries, with zero introns. RET were identified by the criteria of zero introns and an intron-spanning alignment value equal to 1, DUP were defined as duplicated copies originating from their own parental loci, and Unitary pseudogenes were defined as those with a parental source from other gene loci. DUP, Unitary, and RET pseudogenes were further subcategorized into two classes—FULL (alignment coverage ≥ 20%) and TRUNCATED (coverage < 20%)—based on their alignment coverage relative to their respective parental genes.

### 4.4. Multidimensional Analysis of Pseudogene Distribution

We performed a multidimensional analysis of the genomic distribution of pseudogenes across four aspects as follows. For distribution across genomic features, pseudogenes were assigned based on their overlap with exonic, intronic, or intergenic regions using positional annotation, and the results were visualized. Regarding distribution along chromosomes, each chromosome was partitioned into consecutive, non-overlapping 1 Mb windows, with the base-pair coverage of functional genes, transposable elements (TEs), and each pseudogene type calculated per window; these distributions were visualized using Circos (v0.69-8) [55] to generate a circular ideogram, where concentric rings from outer to inner represent the density of functional genes, TEs, all pseudogenes, and the four individual pseudogene types (DUP, FRAG, RET, UNI). For distribution among subgenomes, pseudogenes and their parent genes were assigned to the A, B, or D subgenome based on chromosomal location, and the counts of each pseudogene type across the subgenomes were tallied. Additionally, the distance to parent genes was analyzed: for pseudogene-parent gene pairs located on the same chromosome, the intrachromosomal physical distance between them was calculated, and the distribution of these distances was plotted on a log_2_(Mb + 1) scale.

### 4.5. Enrichment Analysis of Different Types of Pseudogenes

To infer pseudogene functions based on their parent genes, functional annotations of GO and KEGG were performed for all parent genes of wheat pseudogenes using InterProScan (GO) and KoFamScan (KEGG) [56], respectively, and this set of genes served as the background gene set. Subsequently, functional enrichment analysis was conducted on the parental genes of distinct pseudogene types (DUP, RET, UNI). For this analysis, the parental gene sets corresponding to DUP, RET and UNI pseudogenes were treated analogously to differentially expressed gene sets in transcriptome analysis. GO terms and KEGG pathways with a corrected *p* < 0.05 were considered statistically significant.

### 4.6. Association Analysis Between Pseudogenes and Parent Genes

We analyzed pseudogene–parent gene associations from two key dimensions: First, to characterize quantitative relationship within gene families, wheat genes were clustered into gene families using OrthoFinder2 v2.2.6 [57], we then counted the number of functional genes and pseudogenes in each family and visualized their quantitative associations via graphical plotting. Second, for the distribution of sequence similarity, Unitary Pseudogenes (UNI) were excluded, and sequence similarity between the remaining pseudogene types and their parent genes was analyzed using two key metrics—percentage identity and alignment coverage—both extracted from the pseudogene annotation files.

### 4.7. Phylogenetic Analysis of Pseudogene Evolution Dynamics

We conducted three interrelated analyses to characterize the evolutionary dynamics of pseudogenes, including collinearity verification, estimation of formation time, and selection pressure analysis.

First, to construct a set of high-confidence orthologous gene pairs for evolutionary comparison, we performed collinearity analysis. Annotations of protein-coding genes and pseudogenes were merged and partitioned according to the A, B, and D subgenomes. For Unitary and duplicate (DUP) pseudogenes, an all-against-all BLASTP v2.7.1 [58,59,60] search was performed to identify reciprocal best hits (RBHs). Collinear relationships were then identified from these RBH pairs using MCScanX v20200820 [61], with only 1:1 single-copy collinear relationships retained. These filtered orthologous pairs served as the basis for subsequent molecular clock and selection pressure analyses.

Subsequently, it was assumed that sequences of pseudogenes following mutation were not subject to selection pressure. The formation time of these pseudogenes (containing premature termination codons or frameshift mutations) was estimated based on their sequence divergence from parent genes. For each such pseudogene, the genomic sequence downstream of the inactivating mutation was extracted. Sequences shorter than 50 bp or representing less than 50% of the total alignment length were filtered out. Pairwise nucleotide distances (d) between the retained pseudogene sequences and their parent gene CDSs were calculated using the distmat tool from the EMBOSS suite [62]. Divergence time (*T*) was estimated using the formula *T* = *d*/(2*μ*), with a mutation rate (*μ*) of 1.3 × 10^−8^ substitutions per site per year [63]. All pseudogene formation time data were performed using custom scripts written in Python v3.7.9 with pandas [64], numpy [65], and scipy packages [66] for data processing, numerical calculation, and kernel density estimation, respectively. Visualization was implemented via matplotlib v3.7.1 [67].

Finally, selection pressure acting on these orthologous pairs was assessed by calculating the ratio of non-synonymous to synonymous substitution rates (*K*a/*K*s). The coding sequences (CDSs) of each pseudogene–parent gene pair were aligned with MUSCLE. *K*a and *K*s values were then calculated using the yn00 program in the PAML package. The distribution of *K*a/*K*s ratios across different subgenomes and chromosomes was statistically analyzed to infer patterns of selective constraint. Generally, a *K*a/*K*s < 1, =1, or >1 indicates purifying selection, neutral selection, and positive selection, respectively.

### 4.8. Functional Annotation of Pseudogenes and Expression Analysis of Their Parent Genes

For protein domain annotation: The predicted protein sequences of wheat pseudogenes were searched against the Pfam database (https://pfam.xfam.org, accessed on 4 March 2026) using InterProScan v5.59-91.0 [68] to systematically identify conserved domains. The frequency of each domain type was quantified to evaluate functional modules that may have been retained or lost during pseudogenization across the subgenomes.

For tissue-specific expression analysis of parent genes: Based on transcriptomic data from the TriticeaeExpDB database (https://www.triticeaeexpdb.cn, accessed on 4 March 2026), we screened parent genes derived from common wheat with reliable expression (transcripts per kilobase of exon model per million mapped reads [TPM] > 0.5 in at least three samples). The average expression levels of these parent genes across different wheat tissues and developmental stages were calculated, and the values were transformed to log_10_(TPM + 1). Heatmaps were generated to visualize the tissue-specific expression patterns of the parent genes.

## 5. Conclusions

In conclusion, this study presents the first systematic, genome-wide identification and classification of pseudogenes in the A, B, and D subgenomes of common wheat. Our genome-wide analysis reveals that unitary and fragmented pseudogenes are the dominant types in wheat. Transposable elements serve as the core driver of pseudogenization, and our data explain the extremely low proportion of cis-localized pseudogenes in the genome through their mediated mechanisms. The evolution of pseudogenes displays significant asymmetry among the three subgenomes: the B subgenome exhibits higher frequencies of gene loss and pseudogenization events compared to the D subgenome, and a numerically higher (though statistically non-significant) frequency than the A subgenome. This characteristic is associated with the early divergence of its donor species and the consequent accumulation of numerous functionally redundant genes. Meanwhile, wheat pseudogenes do not evolve in a strictly neutral manner but are instead subject to the constraints of purifying selection. The formation of pseudogenes shows three ancient peaks, which may be linked to genomic upheavals experienced by ancestral lineages during different geological periods, rather than being a direct consequence of recent polyploidization. Furthermore, pseudogenization demonstrates clear functional selectivity, with parent genes significantly enriched in functional pathways related to environmental adaptation. This research provides important empirical evidence for deciphering the evolutionary mechanisms of polyploid genomes in wheat and offers a methodological framework and theoretical reference for related studies in other complex polyploid crops.

## Data Availability

The original contributions presented in this study are included in the article/Supplementary Material. Further inquiries can be directed to the corresponding author.

## Supplementary Materials

The following supporting information can be downloaded at https://www.mdpi.com/article/10.3390/plants15050818/s1, Table S1: Master list of genome-wide identified pseudogenes in common wheat; Table S2: Statistical results of pseudogene collinearity types; Table S3: GO enrichment of the parent gene of DUP-type pseudogenes; Table S4: Frequency and annotation of conserved protein domains (Pfam) identified in wheat pseudogenes; Figure S1: Heatmap depicting tissue-specific expression patterns of pseudogene parent genes.

## Abbreviations

The following abbreviations are used in this manuscript:

TE	Transposable Element
MYA	Million Years Ago
WGD	Whole-Genome Duplication
DUP	Duplicate Pseudogenes
UNI	Unitary Pseudogenes
RET	Processed Pseudogenes (Retropseudogenes)
FRAG	Fragmented Pseudogenes
GO	Gene Ontology
KEGG	Kyoto Encyclopedia of Genes and Genomes
*K*a	Non-synonymous substitution rate
*K*s	Synonymous substitution rate
*K*a/*K*s	Ratio of Non-synonymous to Synonymous Substitution Rate
CDS	Coding Sequence
RBH	Reciprocal Best Hits
TPM	Transcripts Per Kilobase of exon model per Million mapped reads

## References

[1] Balfourier F., Bouchet S., Robert S., De Oliveira R., Rimbert H., Kitt J., Choulet F., Paux E. (2019). Worldwide Phylogeography and History of Wheat Genetic Diversity. Sci. Adv..

[2] Sertse D., You F.M., Klymiuk V., Haile J.K., N’Diaye A., Pozniak C.J., Cloutier S., Kagale S. (2023). Historical Selection, Adaptation Signatures, and Ambiguity of Introgressions in Wheat. Int. J. Mol. Sci..

[3] Yue H., Shu D., Wang M., Xing G., Zhan H., Du X., Song W., Nie X. (2018). Genome-Wide Identification and Expression Analysis of the HD-Zip Gene Family in Wheat (*Triticum aestivum* L.). Genes.

[4] Salamini F., Özkan H., Brandolini A., Schäfer-Pregl R., Martin W. (2002). Genetics and Geography of Wild Cereal Domestication in the near East. Nat. Rev. Genet..

[5] Wang X., Chen S., Shi X., Liu D., Zhao P., Lu Y., Cheng Y., Liu Z., Nie X., Song W. (2019). Hybrid Sequencing Reveals Insight into Heat Sensing and Signaling of Bread Wheat. Plant J..

[6] Zhang K., Wang X., Cheng F. (2019). Plant Polyploidy: Origin, Evolution, and Its Influence on Crop Domestication. Hortic. Plant J..

[7] Pingault L., Choulet F., Alberti A., Glover N., Wincker P., Feuillet C., Paux E. (2015). Deep Transcriptome Sequencing Provides New Insights into the Structural and Functional Organization of the Wheat Genome. Genome Biol..

[8] Xie Y., Ying S., Li Z., Zhang Y., Zhu J., Zhang J., Wang M., Diao H., Wang H., Zhang Y. (2023). Transposable Element-Initiated Enhancer-like Elements Generate the Subgenome-Biased Spike Specificity of Polyploid Wheat. Nat. Commun..

[9] Feldman M., Levy A.A. (2005). Allopolyploidy—A Shaping Force in the Evolution of Wheat Genomes. Cytogenet. Genome Res..

[10] Huang Y., Liu Y., Liu C., Yi C., Lai J., Ling H., Su H., Han F. (2025). Distinct Evolutionary Trajectories of Subgenomic Centromeres in Polyploid Wheat. Genome Biol..

[11] Mighell A.J., Smith N.R., Robinson P.A., Markham A.F. (2000). Vertebrate Pseudogenes. FEBS Lett..

[12] Podlaha O., Zhang J. (2010). Pseudogenes and Their Evolution. Encyclopedia of Life Sciences.

[13] Mascagni F., Usai G., Cavallini A., Porceddu A. (2021). Structural Characterization and Duplication Modes of Pseudogenes in Plants. Sci. Rep..

[14] Sisu C., Pei B., Leng J., Frankish A., Zhang Y., Balasubramanian S., Harte R., Wang D., Rutenberg-Schoenberg M., Clark W. (2014). Comparative Analysis of Pseudogenes Across Three Phyla. Proc. Natl. Acad. Sci. USA.

[15] Liu Y.-J., Zheng D., Balasubramanian S., Carriero N., Khurana E., Robilotto R., Gerstein M.B. (2009). Comprehensive Analysis of the Pseudogenes of Glycolytic Enzymes in Vertebrates: The Anomalously High Number of GAPDH Pseudogenes Highlights a Recent Burst of Retrotrans-Positional Activity. BMC Genom..

[16] Xie J., Chen S., Xu W., Zhao Y., Zhang D. (2019). Origination and Function of Plant Pseudogenes. Plant Signal. Behav..

[17] Prade V.M., Gundlach H., Twardziok S., Chapman B., Tan C., Langridge P., Schulman A.H., Stein N., Waugh R., Zhang G. (2018). The Pseudogenes of Barley. Plant J..

[18] Poliseno L., Salmena L., Zhang J., Carver B., Haveman W.J., Pandolfi P.P. (2010). A Coding-Independent Function of Gene and Pseudogene mRNAs Regulates Tumour Biology. Nature.

[19] Cheetham S.W., Faulkner G.J., Dinger M.E. (2020). Overcoming Challenges and Dogmas to Understand the Functions of Pseudogenes. Nat. Rev. Genet..

[20] Sen K., Ghosh T.C. (2013). Pseudogenes and Their Composers: Delving in the “debris” of Human Genome. Brief. Funct. Genom..

[21] Zou C., Lehti-Shiu M.D., Thibaud-Nissen F., Prakash T., Buell C.R., Shiu S.-H. (2009). Evolutionary and Expression Signatures of Pseudogenes in Arabidopsis and Rice. Plant Physiol..

[22] Xu Y.-C., Niu X.-M., Li X.-X., He W., Chen J.-F., Zou Y.-P., Wu Q., Zhang Y.E., Busch W., Guo Y.-L. (2019). Adaptation and Phenotypic Diversification in Arabidopsis Through Loss-of-Function Mutations in Protein-Coding Genes. Plant Cell.

[23] Wang L., Si W., Yao Y., Tian D., Araki H., Yang S. (2012). Genome-Wide Survey of Pseudogenes in 80 Fully Re-Sequenced Arabidopsis Thaliana Accessions. PLoS ONE.

[24] Zhou L., Chen T., Qiu X., Liu J., Guo S. (2023). Evolutionary Differences in Gene Loss and Pseudogenization among Mycoheterotrophic Orchids in the Tribe Vanilleae (*Subfamily vanilloideae*). Front. Plant Sci..

[25] Wicker T., Mayer K.F.X., Gundlach H., Martis M., Steuernagel B., Scholz U., Šimková H., Kubaláková M., Choulet F., Taudien S. (2011). Frequent Gene Movement and Pseudogene Evolution Is Common to the Large and Complex Genomes of Wheat, Barley, and Their Relatives. Plant Cell.

[26] Choulet F., Alberti A., Theil S., Glover N., Barbe V., Daron J., Pingault L., Sourdille P., Couloux A., Paux E. (2014). Structural and Functional Partitioning of Bread Wheat Chromosome 3B. Science.

[27] Xia C., Zhang L., Zou C., Gu Y., Duan J., Zhao G., Wu J., Liu Y., Fang X., Gao L. (2017). A TRIM Insertion in the Promoter of Ms2 Causes Male Sterility in Wheat. Nat. Commun..

[28] Prade V.M. (2018). Pseudogene Dynamics in Plants. Doctoral Dissertation.

[29] Zheng D., Frankish A., Baertsch R., Kapranov P., Reymond A., Choo S.W., Lu Y., Denoeud F., Antonarakis S.E., Snyder M. (2007). Pseudogenes in the ENCODE Regions: Consensus Annotation, Analysis of Transcription, and Evolution. Genome Res..

[30] Balakirev E.S., Ayala F.J. (2003). Pseudogenes: Are They “Junk” or Functional DNA?. Annu. Rev. Genet..

[31] Jia J., Deng P., Li T., Wang K., Gao L., Zhao G., Cui D., Dong Z., Li C., Zhan K. (2025). Transposable Elements Drive the Subgenomic Divergence of Homoeologous Genes to Shape Wheat Domestication and Improvement. Plant Commun..

[32] Charles M., Belcram H., Just J., Huneau C., Viollet A., Couloux A., Segurens B., Carter M., Huteau V., Coriton O. (2008). Dynamics and Differential Proliferation of Transposable Elements during the Evolution of the B and a Genomes of Wheat. Genetics.

[33] Brenchley R., Spannagl M., Pfeifer M., Barker G.L.A., D’Amore R., Allen A.M., McKenzie N., Kramer M., Kerhornou A., Bolser D. (2012). Analysis of the Bread Wheat Genome Using Whole-Genome Shotgun Sequencing. Nature.

[34] Slotkin R.K., Martienssen R. (2007). Transposable Elements and the Epigenetic Regulation of the Genome. Nat. Rev. Genet..

[35] Kim M.Y., Zilberman D. (2014). DNA Methylation as a System of Plant Genomic Immunity. Trends Plant Sci..

[36] Kazazian H.H. (2004). Mobile Elements: Drivers of Genome Evolution. Science.

[37] Kenchanmane Raju S.K. (2021). Epigenomic Atlas in Wheat Reveals Regulatory Elements Specifying Subgenome Divergence. Plant Cell.

[38] Akhunov E.D., Akhunova A.R., Linkiewicz A.M., Dubcovsky J., Hummel D., Lazo G., Chao S., Anderson O.D., David J., Qi L. (2003). Synteny Perturbations between Wheat Homoeologous Chromosomes Caused by Locus Duplications and Deletions Correlate with Recombination Rates. Proc. Natl. Acad. Sci. USA.

[39] Thibaud-Nissen F., Ouyang S., Buell C.R. (2009). Identification and Characterization of Pseudogenes in the Rice Gene Complement. BMC Genom..

[40] Pink R.C., Wicks K., Caley D.P., Punch E.K., Jacobs L., Francisco Carter D.R. (2011). Pseudogenes: Pseudo-Functional or Key Regulators in Health and Disease?. RNA.

[41] Yadav S., Kalwan G., Meena S., Gill S.S., Yadava Y.K., Gaikwad K., Jain P.K. (2023). Unravelling the Due Importance of Pseudogenes and Their Resurrection in Plants. Plant Physiol. Biochem..

[42] Proost S., Pattyn P., Gerats T., Van De Peer Y. (2011). Journey Through the Past: 150 Million Years of Plant Genome Evolution. Plant J..

[43] Ma P.-F., Liu Y.-L., Jin G.-H., Liu J.-X., Wu H., He J., Guo Z.-H., Li D.-Z. (2021). The *Pharus latifolius* Genome Bridges the Gap of Early Grass Evolution. Plant Cell.

[44] Zhang L., Zhu X., Zhao Y., Guo J., Zhang T., Huang W., Huang J., Hu Y., Huang C.-H., Ma H. (2022). Phylotranscriptomics Resolves the Phylogeny of Pooideae and Uncovers Factors for Their Adaptive Evolution. Mol. Biol. Evol..

[45] Marcussen T., Sandve S.R., Heier L., Spannagl M., Pfeifer M., Jakobsen K.S., Wulff B.B.H., Steuernagel B., Mayer K.F.X., The International Wheat Genome Sequencing Consortium (2014). Ancient Hybridizations Among the Ancestral Genomes of Bread Wheat. Science.

[46] Gill B.S., Appels R., Botha-Oberholster A.-M., Buell C.R., Bennetzen J.L., Chalhoub B., Chumley F., Dvořák J., Iwanaga M., Keller B. (2004). A Workshop Report on Wheat Genome Sequencing. Genetics.

[47] Zhu T., Wang L., Rimbert H., Rodriguez J.C., Deal K.R., De Oliveira R., Choulet F., Keeble-Gagnère G., Tibbits J., Rogers J. (2021). Optical Maps Refine the Bread Wheat *Triticum aestivum* Cv. Chinese Spring Genome Assembly. Plant J..

[48] Bairoch A. (1996). The SWISS-PROT Protein Sequence Data Bank and Its New Supplement TREMBL. Nucleic Acids Res..

[49] Bao W., Kojima K.K., Kohany O. (2015). Repbase Update, a Database of Repetitive Elements in Eukaryotic Genomes. Mob. DNA.

[50] Mistry J., Chuguransky S., Williams L., Qureshi M., Salazar G.A., Sonnhammer E.L.L., Tosatto S.C.E., Paladin L., Raj S., Richardson L.J. (2021). Pfam: The Protein Families Database in 2021. Nucleic Acids Res..

[51] Li W., Godzik A. (2006). Cd-Hit: A Fast Program for Clustering and Comparing Large Sets of Protein or Nucleotide Sequences. Bioinformatics.

[52] Buchfink B., Xie C., Huson D.H. (2015). Fast and Sensitive Protein Alignment Using DIAMOND. Nat. Methods.

[53] Birney E., Clamp M., Durbin R. (2004). GeneWise and Genomewise. Genome Res..

[54] Pearson W.R. (2013). An Introduction to Sequence Similarity (“Homology”) Searching. Curr. Protoc. Bioinform..

[55] Krzywinski M., Schein J., Birol I., Connors J., Gascoyne R., Horsman D., Jones S.J., Marra M.A. (2009). Circos: An Information Aesthetic for Comparative Genomics. Genome Res..

[56] Aramaki T., Blanc-Mathieu R., Endo H., Ohkubo K., Kanehisa M., Goto S., Ogata H. (2020). KofamKOALA: KEGG Ortholog Assignment Based on Profile HMM and Adaptive Score Threshold. Bioinformatics.

[57] Emms D.M., Kelly S. (2019). OrthoFinder: Phylogenetic Orthology Inference for Comparative Genomics. Genome Biol..

[58] Altschul S.F., Gish W., Miller W., Myers E.W., Lipman D.J. (1990). Basic Local Alignment Search Tool. J. Mol. Biol..

[59] Altschul S. (1997). Gapped BLAST and PSI-BLAST: A New Generation of Protein Database Search Programs. Nucleic Acids Res..

[60] Camacho C., Coulouris G., Avagyan V., Ma N., Papadopoulos J., Bealer K., Madden T.L. (2009). BLAST+: Architecture and Applications. BMC Bioinf..

[61] Wang Y., Tang H., DeBarry J.D., Tan X., Li J., Wang X., Lee T., Jin H., Marler B., Guo H. (2012). MCScanX: A Toolkit for Detection and Evolutionary Analysis of Gene Synteny and Collinearity. Nucleic Acids Res..

[62] Rice P., Longden I., Bleasby A. (2000). EMBOSS: The European Molecular Biology Open Software Suite. Trends Genet..

[63] SanMiguel P., Gaut B.S., Tikhonov A., Nakajima Y., Bennetzen J.L. (1998). The Paleontology of Intergene Retrotransposons of Maize. Nat. Genet..

[64] McKinney W. Data Structures for Statistical Computing in Python. Proceedings of the 9th Python in Science Conference.

[65] Harris C.R., Millman K.J., Van Der Walt S.J., Gommers R., Virtanen P., Cournapeau D., Wieser E., Taylor J., Berg S., Smith N.J. (2020). Array Programming with NumPy. Nature.

[66] Virtanen P., Gommers R., Oliphant T.E., Haberland M., Reddy T., Cournapeau D., Burovski E., Peterson P., Weckesser W., Bright J. (2020). SciPy 1.0: Fundamental Algorithms for Scientific Computing in Python. Nat. Methods.

[67] Hunter J.D. (2007). Matplotlib: A 2D Graphics Environment. Comput. Sci. Eng..

[68] Jones P., Binns D., Chang H.-Y., Fraser M., Li W., McAnulla C., McWilliam H., Maslen J., Mitchell A., Nuka G. (2014). InterProScan 5: Genome-Scale Protein Function Classification. Bioinformatics.

