# Supplementary figures and images for: A Genome-Wide Pseudogene Map Reveals the Asymmetric Evolution of the A, B, and D Subgenomes in Common Wheat
Source: Plants (Basel). 2026 Mar 7;15(5):818. doi: 10.3390/plants15050818 (PMC12987264; doi:10.3390/plants15050818)

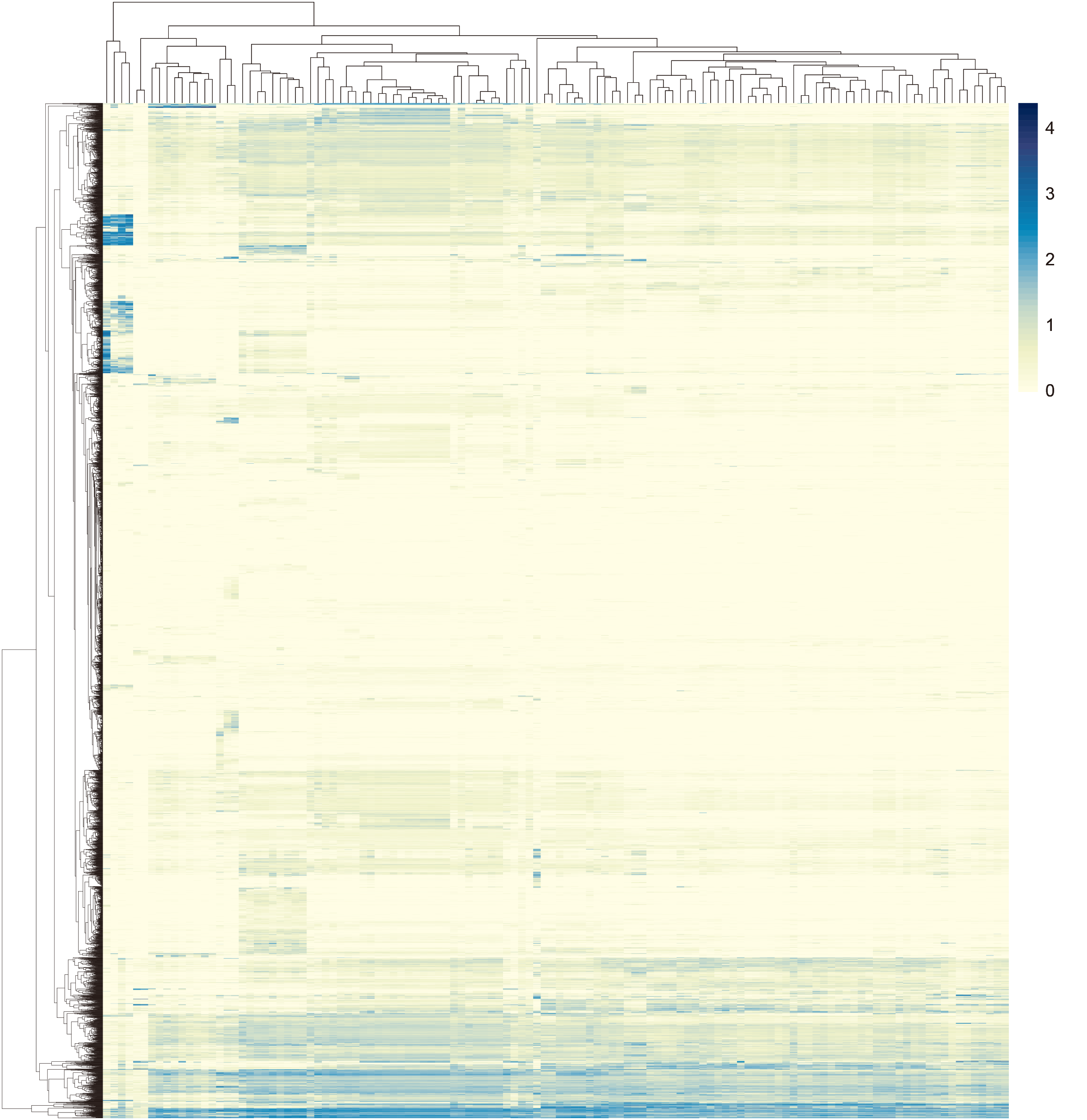

Supplement: Supplementary file 1 [file plants-15-00818-s001.zip › Figure S1.png]
